# Phylogenetic Characteristics of Canine Parvovirus Type 2c Variant Endemic in Shanghai, China

**DOI:** 10.3390/v13112257

**Published:** 2021-11-10

**Authors:** Chengqian Liu, Jun Gao, Hong Li, Fengping Sun, Hongyu Liang, Huili Liu, Jianzhong Yi

**Affiliations:** 1 Institute of Animal Husbandry and Veterinary Science, Shanghai Academy of Agricultural Sciences, Shanghai 201106, China; liuchengqian@saas.sh.cn (C.L.); gjsaas@gmail.com (J.G.); lihong20061029@163.com (H.L.); sfp_hot@163.com (F.S.); 2 National Demonstration Center for Experimental Fisheries Science Education, Shanghai Ocean University, Shanghai 201306, China; LHY4830@163.com

**Keywords:** canine parvovirus, CPV-2, VP2 capsid protein

## Abstract

Canine parvovirus type 2 (CPV-2) has spread and mutated globally over the past 40 years. In the present study, 206 samples from dogs suspected of CPV-2 infection were collected from five veterinary clinics in Shanghai city, China. The average positive rate for CPV-2 was detected to be 40.78% using the PCR method. Using an F81 cell (feline kidney cell) culture, the isolates of three CPV-2c strains were obtained. The near full-length genome sequences of the isolates were determined and submitted to GenBank: CPV-SH2001 (MW650830), CPV-SH2002 (MW811188), and CPV-SH2003 (MW811189). By comparing the amino acid sequences of 12 CPV strains with those of 48 related strains retrieved from GenBank, all of the CPV strains from Shanghai were typed as belonging to a relatively new CPV-2c variant spreading in Asia, with typical amino acid residues (5Gly, 267Tyr, 324Ile, and 370Arg) in the VP2 protein. The divergence time of this new CPV-2c clade was estimated by the phylogenetic tree using the maximum likelihood and RelTime with Dated Tips (RTDT) approaches. Our results indicate that the 426 and 324 VP2 amino acid residues are under strong selection pressure with a posterior probability of 0.966 and 0.943, respectively. Therefore, this study provides insight into the phylogenetic characteristics of the current CPV-2c variant in Shanghai city, China.

## 1. Introduction

Canine parvovirus (CPV), which belongs to the genus *Protoparvovirus*, family *Parvoviridae*, emerged in 1978–1979 as the causative agent of a new enteric and myocardial disease in dogs and has sustained its spread globally [1,2,3,4]. Phylogenetic analysis shows that all CPV isolates descended from a single ancestor considered to be a change in the host range due to mutations in feline panleukopenia virus (FPV) [4,5]. CPV is a single-stranded DNA virus involving a genome with two open reading frames (ORFs). The first ORF encodes two non-structural proteins: NS1 and NS2. The second ORF encodes two structural (capsid) proteins: VP1 and VP2 [6]. The VP2 capsid protein is the major antigenic protein, with most nonsynonymous substitutions occurring in this region, which determines viral tissue tropism and host range [3,7].

CPV-2 was named to distinguish it from the unrelated canine minute virus (CnMV) or canine parvovirus type 1 (CPV-1) [8,9]. Earlier studies revealed that CPV-2 was replaced in the United States around 1980 by an antigenically and genetically variant virus named CPV-2a [10]. Since 1986, another virus, named CPV-2b, has largely replaced CPV-2a among virus isolates [11]. As it has continuous antigenic drift, a newer variant, CPV-2c, was reported from Italy in 2001 [12], with the amino acid (aa) mutation of Asp426Glu in the VP2 gene. According to the investigation, the proportion of different variants in China has changed in recent years, and CPV-2c appears to be growing rapidly [13]. Moreover, neighboring Asian countries such as Mongolia [14] and Vietnam [15] have reported a high proportion of CPV-2c-positive samples collected from 2016 to 2018. A CPV-2c variant with typical aa residues (5Gly, 267Tyr, 324Ile, and 370Arg) in the VP2 protein has been recognized as the “Asian CPV-2c” genotype [16,17,18,19]. This genomic variant was never reported prior to 2013 [15] and has even reached Italy and Nigeria in recent years [20,21,22].

Shanghai, a mega city in eastern China with a resident population of over 24 million, has seen an explosive growth in pet ownership in the last decade. Concerns have been raised about the level of protection provided by the CPV-2-attenuated vaccine due to the emergence of new variant strains such as CPV-2c. In this study, we aimed to investigate the prevalence of CPV-2 in domestic dogs and the phylogenetic characteristics of the virus variants in Shanghai, China. 

## 2. Materials and Methods

### 2.1. Sample Collection

In this study, 206 fecal samples from dogs with typical clinical signs of CPV-2 infection [1,2] were collected from January to October 2020 from five veterinary clinics in four districts (Minhang, Jiading, Pudong, and Xuhui) of Shanghai, China. All samples were collected with the pet owners’ consent, following the recommendations in the Guide for the Care and Use of Laboratory Animals of the Ministry of Science and Technology of the People’s Republic of China. This study was reviewed and approved by the Experimental animal Ethics committee of the Shanghai Academy of Agricultural Sciences.

### 2.2. Viral DNA Extraction and Amplification

Virus DNA extraction kits (Takara^®^ Beijing, China) were used to extract the total viral DNA from fecal samples according to the manufacturer’s instructions. The extracted DNA was used for the detection of CPV-2 using the polymerase chain reaction (PCR) method using a primer pair (P1: 5′-GAATCTGCTACTCAGCCAAC-3′ and P2: 5′-GTGCACTATACCACCACCTCAGC-3′), according to the China National Standards’ “Diagnostic techniques for canine parvovirus disease” (GB/T 27533-2011). The PCR-positive samples showed a band at 559 base pairs. Next, 12 positive samples were randomly selected to amplify the full-length VP2 gene by using two primer pairs listed in Table 1 in Supplementary Data S2. The PCR reaction included 2.0 μL of template, 1.0 μL each of primer (10 μmol/L), 12.5 μL of 2 × Phanta Max Master mix (Vazyme Biotech Co., Ltd. (Nanjing, Jiangsu, China), and sufficient ddH_2_O to increase the volume to 25 μL. Amplification was carried out as follows: one cycle at 95 °C for 5 min, followed by 35 cycles at 95 °C for 15 s, 56 °C for 15 s, 72 °C for 50 s, and extension at 72 °C for 5 min. PCR products were detected by electrophoresis in 1.2% agarose gels.

### 2.3. Virus Isolation

Three CPV-2-positive samples were immersed in serum-free DMEM medium (Hyclone™) and centrifuged at 10,000 rpm for 10 min, and then the supernatant was filtered through a 0.22 μm Millipore filter (Millipore^®^, Burlington, MA, USA) and inoculated into the F81 (feline kidney cell) cell monolayer at 37 °C under 5% CO_2_. The F81 cell line CL-0081 was kindly provided by Procell Life Science & Technology Co., Ltd. (Wuhan, Hubei, China). The culture supernatants were harvested when the typical cytopathic effect was observed in almost 85% of the cells, and, subsequently, the culture medium was frozen at −80 °C.

### 2.4. Immunofluorescence Assay

The F81 cells were seeded in 96-well plates (10^4^ cells/well, triplicates). The three CPV-2 isolates were inoculated and incubated for 36 h at 37 °C under 5% CO_2_. Thereafter, the DMEM medium was removed, and the cells were washed with phosphate-buffered saline (PBS) for three rounds, followed by 75% ethanol and 1% Triton-X100 fixation for 15 min. The primary monoclonal antibody 5B10 against CPV provided by Biocare Diagnostics Ltd. (Zhuhai, Guangdong, China) was incubated for 1 h at 37 °C, the secondary antibody FITC AffiniPure Goat Anti-Mouse IgG (H+L) containing fluorescein isothiocyanate provided by Amyjet Scientific Co., Ltd. (Wuhan, Hubei, China) was incubated for 2 h, and the fixed cells were washed using PBS for three cycles. Finally, an inverted fluorescence microscope was used to analyze the fluorescence of the cells; normal F81 cells were used as negative controls.

### 2.5. Near Full-Length Genome Sequencing of CPV-2 Isolates

According to the CPV genome sequence (accession number MG013488) registered in GenBank, five primer pairs (Table 2 in Supplementary Data S2) were designed to amplify the near full-length sequences, which were obtained through PCR amplification, Sanger sequencing, and sequence assembly. The PCR reaction system was 50 μL: 3 μL of DNA template, 5 μL of 10 × buffer (Mg^2+^ free), 4 μL of MgSO_4_ (25 mM), 5 μL of dNTPs (2 mM), 1.5 μL of upstream and downstream primers (10 μM), 2 μL of KOD plus enzyme (Toyobo Biotechnology), and 28 μL of ddH_2_O. PCR amplification was achieved using 36 cycles of denaturation at 94 °C for 30 s, annealing at 58 °C for 30 s, and polymerization at 68 °C for 3 min. After electrophoresis in a 1.2% agarose gel and ethidium bromide staining, the PCR products were sent to BioSune Biotechnology Co., Ltd. (Shanghai, China) for sequencing.

### 2.6. Phylogenetic Analysis

The phylogenetic tree was inferred based on the complete VP2 aa sequences (1755bp, 584 aa) using the maximum likelihood method approaches implemented in MEGA X [23] with 1000 bootstrap replicates, and the cut-off value for the condensed tree was 50%. The Jones–Taylor–Thornton (JTT) model with a discrete gamma (G) distribution obtained the lowest Bayesian information criterion (BIC) scores and was set as the nucleotide substitution model. The 12 CPV-2 full-length VP2 aa sequences obtained in this study were aligned along with those of 48 related strains, including feline panleukopenia virus (FPV) and all subtypes of CPV-2/-2a/-2b/-2c sequences retrieved from GenBank. We re-labeled the names of all VP2 sequences using the GenBank accession number, the variant type of the virus, the country (region), and the year of collection (Supplementary Data S1).

For the divergence time estimation, a time tree was computed in MEGA X where the divergence time was estimated for all branching points in a tree using the RelTime with Dated Tips (RTDT) method [24]. This method does not require assumptions for lineage rate variation and is suitable for the analysis of DNA and protein sequences from fast-evolving pathogens [24]. Two FPV sequences were designated as an outgroup taxon, and for all sequences, the reported years of sampling dates registered in GenBank were used as the tip dates (sample times) for calibration constraints. A Bayesian analysis using the MrBayes v3.2.7 software [25] based on the model (JC69+G+I) with Markov chain Monte Carlo (MCMC) was run for 1,300,000 generations; samplefreq and sump burnin, set to 1000 and 325, respectively, were also performed. The FigTree v1.4.4 software was used to display the Bayesian phylogenetic tree.

### 2.7. Structural Analysis of VP2 Protein

The protein structure analysis was performed using the VP2 aa sequence of the Shanghai CPV-2c isolate (MW811189). First, we implemented protein BLAST (Basic Local Alignment Search Tool) with the Protein Data Bank (PDB) database [26] using the blastp program on the National Center for Biotechnology Information website (https://www.ncbi.nlm.nih.gov/, accession date, 16 August 2021). By calculating the score and the coverage, PDB: 1C8H_A [27,28] (Canine Parvovirus Strain D Empty Capsid Structure at Ph 5.5) was downloaded as the template for 3D protein modeling. Next, we constructed 3D CPV-2 models by performing homology modeling using the MODELLER software v10.1 (https://salib.org/modeller/, accession date, 19 August 2021) [29]. Finally, the visualization and labeling of the VP2 protein tertiary structure were carried out using the PyMOL software v2.5 [30].

### 2.8. Selection Pressure Site Analysis

To detect non-neutral selection sites, we used the fast unconstrained Bayesian approximation (FUBAR) [31] and fixed effects likelihood (FEL) [32] methods in the Datamonkey program (https://www.datamonkey.org/, accession date, 7 September 2021) to analyze the sites in the CPV-2 VP2 aa sequences under positive selection. FUBAR employs a Bayesian algorithm to infer rates, with a posterior probability of >0.9 strongly suggesting positive selection [31]. FEL uses a maximum likelihood (ML) approach to infer nonsynonymous (dN) and synonymous (dS) substitution rates on a per-site basis for a given coding alignment and corresponding phylogeny [32].

## 3. Results

### 3.1. Positive Detection Rate of CPV-2 in Canine Patients

**Figure 1:**
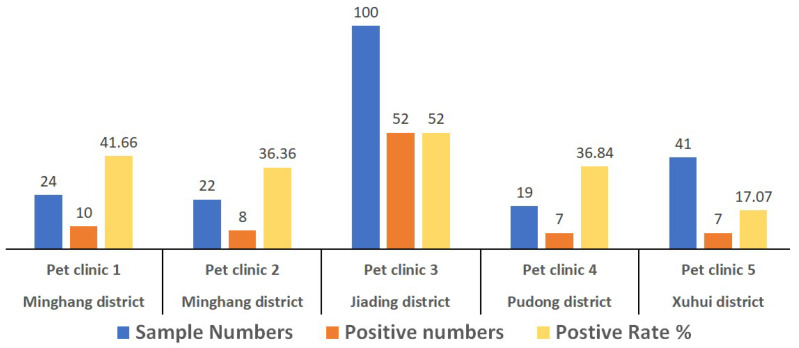
Positive detection rate of CPV-2 in canine patients. A total of 206 fecal specimens from canine patients collected from five pet clinics in four urban districts of Shanghai (Minhang, Jiading, Pudong, and Xuhui) were tested for CPV-2 using the PCR method.

For this study, a total of 206 fecal specimens from dogs suspected of CPV infection were collected from Shanghai, China. The results show that the CPV-2-positive rate among the pet clinics ranged from 17.07% to 52% (Figure 1), based on the PCR method. The overall positive rate was 40.78% (84/206). Based on the analysis of the full-length VP2 gene sequences, the viral strains of 12 positive samples were typed as CPV-2c variants.

### 3.2. CPV Isolation and Identification

**Figure 2:**
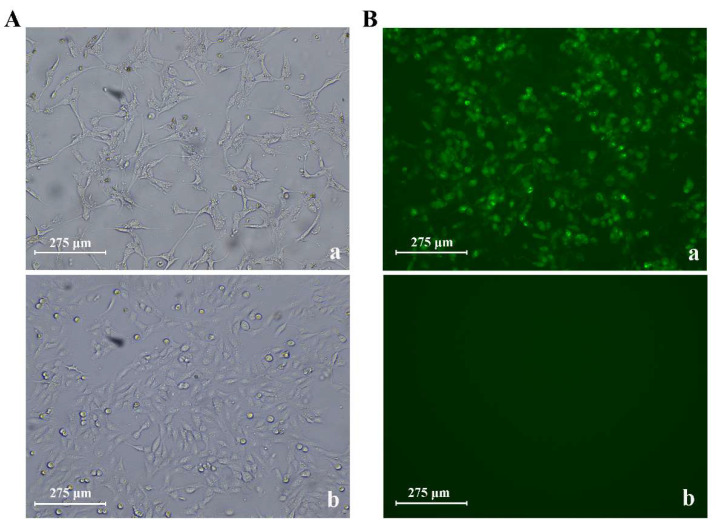
CPV-2 isolation and proliferation. (**A**) Typical cytopathic morphological changes in CPV-2-infected F81 cells (**a** in **A**) compared with the normal F81 control cells (**b** in **A**). (**B**) The immunofluorescence assay of the CPV-2-infected cells (**a** in **B**) and the normal F81 control cells (**b** in **B**).

Three CPV-2 isolates were obtained from the fecal samples through virus isolation and cell culture. CPV-infected cells showed typical cytopathic morphological changes compared to the cells in the control group (Figure 2A). Virus isolation was confirmed by an immunofluorescence assay (Figure 2B). After sequencing and assembling, we obtained near full-length genomic information of the three CPV-2 isolates: CPV-SH2001 (accession number: MW650830), CPV-SH2002 (accession number: MW811188), and CPV-SH2003 (accession number: MW811189). The sequence fragment contains the full-length (4269bp) coding region of CPV-2, including the complete NS1, NS2, VP1, and VP2 genes. The homology of the coding region of the three isolates was greater than 99.8%.

### 3.3. Phylogenetic Tree and Divergence Time Estimate

**Figure 3:**
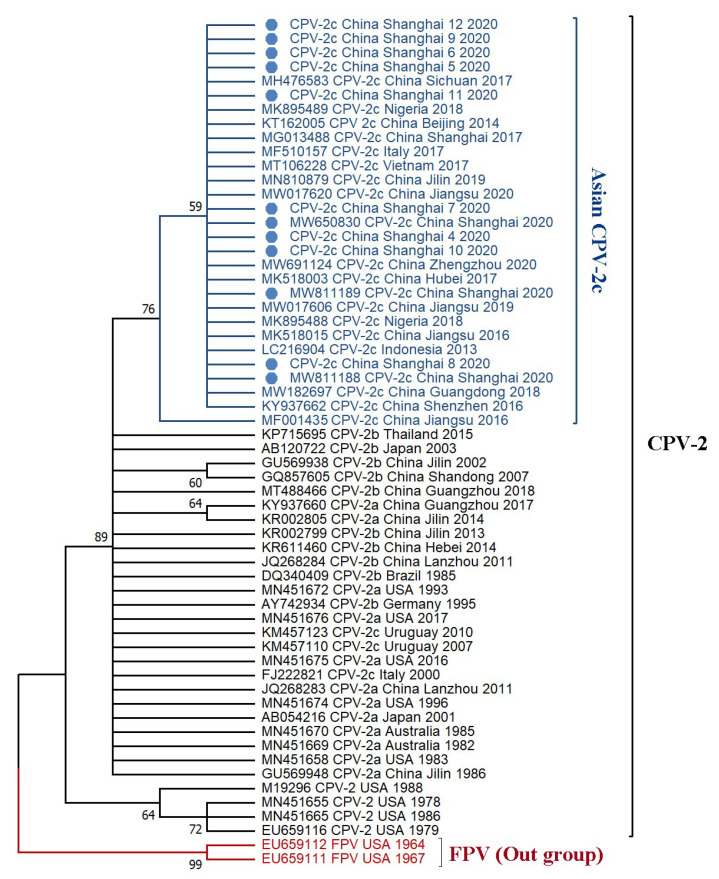
Phylogenetic analysis based on complete VP2 amino acid sequences. The phylogenetic tree was inferred using the maximum likelihood method (JTT+G substitution model) conducted in MEGA X [23] with 1000 bootstrap replicates, with the cut-off value for the condensed tree at 50%. The red clade includes two FPV sequences, the black clade contains all the CPV-2 variants, and the blue clade indicates the new “Asian CPV-2c” clade.

The results reveal that all of the CPV-2 variants originated from FPV, with 99% bootstrap confidence. For CPV-2, all of the CPV-2a, -2b, and -2c variants originated from earlier CPV-2 USA strains (1978–1988). The sequences of the CPV-2c strains from Shanghai cluster in the phylogenetic tree along with other CPV-2c strains from Asia, in a clade depicted as “Asian CPV-2c” (Figure 3). Topologically consistent phylogenetic trees with high posterior probability support were also obtained using Bayesian methods (Supplementary Data S2).

**Figure 4:**
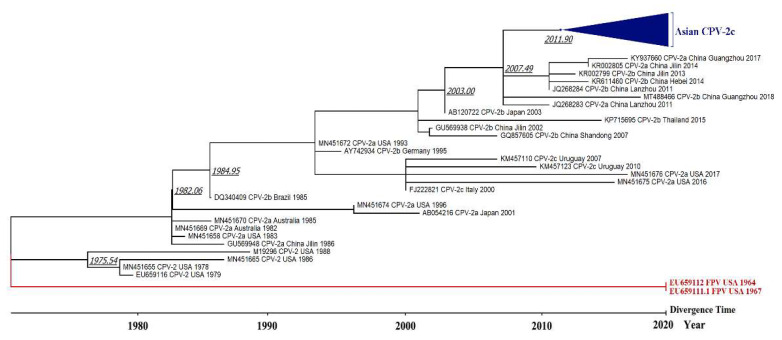
Time tree to infer the divergence time of CPV-2 variants. The time tree was calculated in MEGA X where divergence time was estimated using the RelTime with Dated Tips (RTDT) method [23,24]. Two FPV sequences (red color) were designated as an outgroup taxon and all sequences used the year of sampling dates as the tip dates for calibration constraints. Italicized underlined numbers at the branches indicate the divergence time estimated by the calculation. The divergence time of the Asian CPV-2c clade was estimated to be between 2011 and 2013.

In addition, we calculated the time tree using the RTDT method to investigate the divergence time of the CPV-2 variants. The results reveal that CPV-2 diverged from FPV around the 1970s, and the new “Asian CPV-2c” clade was differentiated between 2011 and 2013 (Figure 4).

### 3.4. Amino Acid Site Analysis of the VP2 Gene

**Figure 5:**
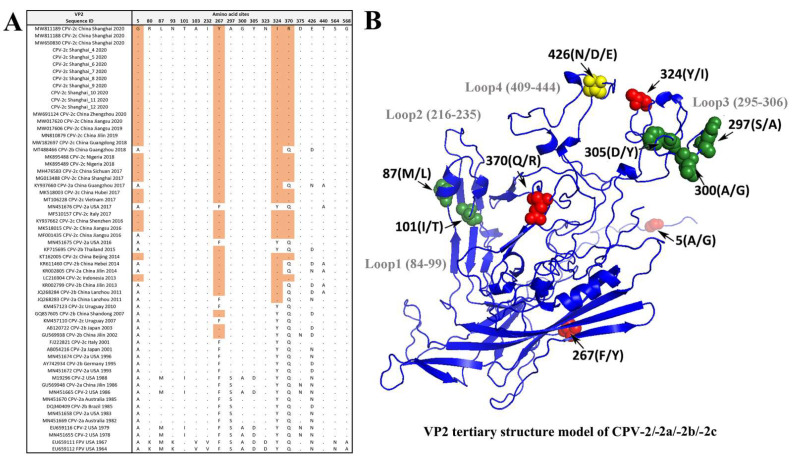
The VP2 variants and structure between the FPV and CPV-2/-2a/-2b/-2c variants. (**A**) The variants and amino acid variation sites in the VP2 gene. (**B**) A VP2 tertiary model of the variants. Amino acids are represented by single-letter abbreviations. The site at position 426 (yellow color), located at the top of the protrusion region of the loop 4 structure [27], is the signature site that distinguishes CPV-2a (Asn), -2b (Asp), and -2c (Glu). The five specific sites at 87Met, 101Ile, 297Ser, 300Ala, and 305Asp for CPV-2 variants were labeled with green color in B. The characteristic mutations of 5Gly, 267Tyr, 324Ile, and 370Arg in the new Asian CPV-2c variant are marked with red color in (**B**).

The VP2-426 position is the signature site that can type the CPV genotype: CPV-2a (Asn), -2b (Asp), and -2c (Glu) variants. The results show that 12 Shanghai CPV-2 strains in this study were characterized with the Glu amino acid at the VP2-426 site, indicating that they are all CPV-2c variants. The protein structure analysis also revealed several variation sites located in the loop structure region of the VP2 protein, such as sites 426/440 located at loop 4, and sites 297/300/305 located at loop 3. Further analysis indicated that all 12 Shanghai CPV-2 strains belong to a new CPV-2c genomic variant with typical amino acid (5Gly, 267Tyr, 324Ile, and 370Arg) residues in the VP2 protein, which differs from the earlier CPV-2c variant (FJ222821 CPV-2c Italy 2001 and KM457110 CPV-2c Uruguay 2007) (Figure 5).

### 3.5. Positive Selection Pressure Sites

The positive selection sites were evaluated using the FUBAR and FEL methods. The FUBAR analysis strongly suggested positive selection (adaptive molecular evolution) at 2 sites: 426 and 324, with a posterior probability of 0.966 and 0.943, respectively (Table 1), and 40 negative selection sites. The FEL method detected pervasive positive selection at 0 sites and pervasive negative selection at 38 sites with a p-value threshold of 0.1 (Supplementary Data S3).

## 4. Discussion

Despite the widespread use of the CPV-2 vaccine, the variants continue to spread and mutate in canines [3]. In the present study, 206 fecal samples from dogs suspected of CPV-2 infection collected from five pet clinics were found to be CPV-2 positive at an average rate of 40.78%, which indicates that CPV-2 remains one of the most important infectious agents found among domestic dogs in Shanghai, China. For this reason, it is becoming increasingly important to have timely information on the genetic variation of locally prevalent variants.

Several previous studies have confirmed that host ranges, antigenicity, and hemagglutination properties are controlled by the VP2 capsid protein of CPV-2 [5,33,34]. As can be seen from the 3D structure of the VP2 protein, residue 426 of VP2 is located in the outermost part of the threefold axis of the loop 4 structure at the site of greatest antigenicity of the virus [27,35]. The CPV-2c variant, which was reported in Italy in 2001 [12], is characterized by the affected position 426 (Asp to Glu), located at a major antigenic site of the viral capsid. The present study identified that all 12 Shanghai CPV-2 isolates are CPV-2c variants with Glu amino acid residues at position 426 of the VP2 protein. Moreover, our results indicate that the 426 and 324 VP2 amino acid residues are under strong positive selection with a posterior probability of 0.966 and 0.943, respectively. Residue 324 is adjacent to site 323 on the loop 3 structure, which has been reported together with residue 93 to play an important role in binding to the canine transferrin receptor and affect the virus host range [4].

In recent years, a CPV-2c genomic variant, characterized by specific amino acid residues in the VP2 protein (5Gly, 267Tyr, 324Ile, and 370Arg), has emerged in Asia and, more recently, spread in Europe and Africa [16,17,18,19]. Indeed, this CPV-2c variant with Asian origins was described in Italy and Nigeria and, in at least one of these cases, its spread was associated with the introduction of a dog from Asia [20,21,22]. However, our results show that 267Tyr and 324Ile not only appear in the latest Asian CPV-2c variants but are already present in some Asian CPV-2a or CPV-2b variants such as JQ268283 CPV-2a China Lanzhou 2011 and AB120722 CPV-2b Japan 2003. This study estimated the divergence time of this new CPV-2c genomic variant to be between 2011 and 2013. A recent study on the typing of the CPV-2 strains circulating in East China from 2018 to 2020 showed a high positive rate of 77.19% (44/57) for CPV-2c variants, and, among them, all 14 CPV-2 isolates from Shanghai (100%) were typed as new CPV-2c variants [19]. Our results are consistent with these data, indicating this CPV-2c variant as the current dominant genotype circulating in Shanghai, China.

Although it is unclear whether these key mutations have an impact on the infectivity and pathogenicity of the virus, other authors hypothesized that the new type “Asian CPV-2c” is more virulent compared with previous viruses [18]. Studies have reported that vaccination with canine parvovirus type 2 (CPV-2) protects against challenge with CPV-2c isolates [36,37]. However, an outbreak of CPV-2c infection has also been reported in vaccinated adult dogs [38]. It is critical to conduct an evaluation of the protection efficiency of existing commercial CPV-2 vaccines against this new “Asian CPV-2c” variant. In addition, enhanced epidemiological surveillance of CPV-2 genomic variants will facilitate understanding of antigen drift and provide effective prevention and control measures.

## 5. Conclusions

CPV-2c variants have spread worldwide and become the dominant genotype of CPV-2 in domestic dogs in Shanghai, China. Genetic mutation and structural analyses of the VP2 capsid protein indicate that a new type of “Asian CPV-2c” variant, possessing four key aa site mutations at 5Gly, 267Tyr, 324Ile, and 370Arg, diverged from earlier reported -2c variants between 2011 and 2013 and, therefore, deserves further attention. The results reveal that aa sites at positions 426 and 324 of VP2 are under selective pressure, and their location in the tertiary structure of the VP2 protein also demonstrates the importance of these aa mutation sites. Regular pathogenetic surveillance of prevalent variants of CPV-2 needs to be enhanced.

## Figures and Tables

**Table 1:** The sites with a tendency to be under positive selection pressure.

Site	α	β	β-α	Prob [α > β]	Prob [α < β]	BayesFactor [α < β]
426	3.971	46.531	42.560	0.005	0.966 ^1^	45.222
324	3.948	35.271	31.323	0.024	0.943 ^1^	26.092
375	4.632	23.224	18.591	0.066	0.892	13.063
440	2.471	14.691	12.219	0.079	0.885	12.114
13	2.946	15.705	12.759	0.084	0.878	11.339
267	4.632	14.76	10.128	0.126	0.828	7.613
87	1.072	5.364	4.293	0.222	0.733	4.332
370	3.086	6.645	3.559	0.267	0.684	3.419
579	2.298	3.554	1.255	0.296	0.652	2.969
5	2.297	3.484	1.187	0.297	0.651	2.949

^1^ Sites 426 and 324 with posterior probabilities > 0.9 are strongly suggestive of positive selection in FUBAR analysis.

## Data Availability

Three CPV-2c near full-length sequences from this study were deposited in the Genbank (https://www.ncbi.nlm.nih.gov/genbank/) under accession number of MW650830, MW811188, and MW811189.

## Supplementary Materials

The following are available online at https://www.mdpi.com/article/10.3390/v13112257/s1, Data S1: 60 sequences of information from the VP2 gene. Data S2: Primers and Bayesian phylogenetic tree. Data S3: The analysis of selection pressure sites in VP2 sequences.
