# Supplementary material for: Phylogenetic Characteristics of Canine Parvovirus Type 2c Variant Endemic in Shanghai, China
Source: Viruses. 2021 Nov 10;13(11):2257. doi: 10.3390/v13112257 (PMC8618335; doi:10.3390/v13112257)
Supplement: Supplementary file 1 [file viruses-13-02257-s001.zip › DATA S2. Primers and BI Tree.pdf]

Table S1. Primers used for the amplification of CPV-VP2 gene

Name	Sequence (5′-3′)	Product (bp)	Position (nt)[a]
VP2-F1	AGAGACAATCTTGCACCAAT	1068	2766-3832
VP2-R1	TGTGTAGACGCCTCAAAAGAATAAT		
VP2-F2	GAACAGGTGATGAATTTGCTACAGG	1209	3550-4758
VP2-R2	CAACCACCCACACCATAAC		

[a] Oligonucleotide positions are referred to the genomic sequences of CPV-2c strain (accession number:MG013488)

Table S2. Primers used for the amplify the near full-length sequence of CPV-2

Name	Sequence (5′-3′)	Product (bp)	Position (nt)[a]
CPV-F1	ATAGACCGTTACTGACATTCG	1022	205-1226
CPV-R1	TCCTGGTTGTGCCATCATTTC		
CPV-F2	CCAGAAACCGTTGAAACCACAG	937	1044-1980
CPV-R2	GTATTTTAGGCTCCGCCCAGTT		
CPV-F3	GGGAAAAGTACCAGAATGGGATG	974	1934-2907
CPV-R3	TAGAAATCCCCACACCCCCAG		
CPV-F4	GCACCAATGAGTGATGGAGGAGT	1056	2778-3832
CPV-R4	TGTGTAGACGCCTCAAAAGAATAAT		
CPV-F5	GAACAGGTGATGAATTTGCTACAGG	1209	3550-4758
CPV-R5	CAACCACCCACACCATAAC		

[a] Oligonucleotide positions are referred to the genomic sequences of CPV-2c strain (accession number:MG013488)

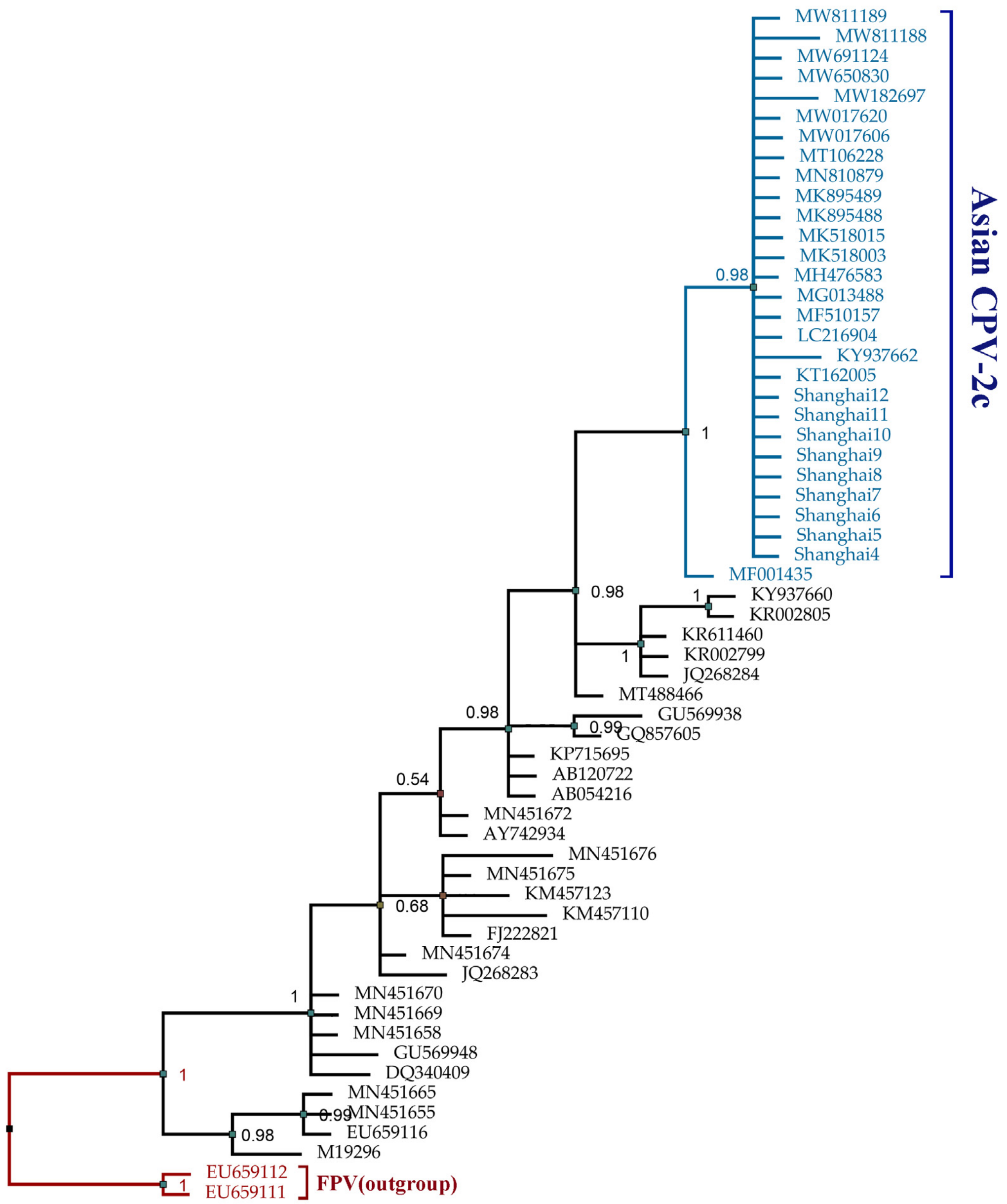

Figure S1. The Bayesian phylogenetic tree inferred based on 60 VP2 sequences

MrBayes v3.2.7 software was used to infer the phylogenetic tree on the model (JC69+G+I) with Markov chain Monte Carlo (MCMC) run for 1,300,000 generations, samplefreq and sump burnin set to 1000 and 325, respectively. The FigTree v1.4.4 software was used to display this Bayesian phylogenetic tree after the analysis. The Arabic numbers at the nodes on the graph represent supported by the percentage of Bayesian posterior probabilities (BPP).
